# Repurposing Drugs in Oncology (ReDO)—clarithromycin as an anti-cancer agent

**DOI:** 10.3332/ecancer.2015.513

**Published:** 2015-02-24

**Authors:** An MT Van Nuffel, Vidula Sukhatme, Pan Pantziarka, Lydie Meheus, Vikas P Sukhatme, Gauthier Bouche

**Affiliations:** 1 Anticancer Fund, Brussels, 1853 Strombeek-Bever, Belgium; 2 GlobalCures, Inc, Newton, MA 02459, USA; 3 The George Pantziarka TP53 Trust, London KT1 2JP, UK; 4 Beth Israel Deaconess Medical Centre and Harvard Medical School, Boston, MA 02215, USA

**Keywords:** ReDO project, drug repositioning, clarithromycin, neoplasms, antineoplastic agents, anti-bacterial agents

## Abstract

Clarithromycin (CAM) is a well-known macrolide antibiotic available as a generic drug. CAM is traditionally used for many types of bacterial infections, treatment of Lyme disease and eradication of gastric infection with *Helicobacter pylori*. Extensive preclinical and clinical data demonstrate a potential role for CAM to treat various tumours in combination with conventional treatment. The mechanisms of action underlying the anti-tumour activity of CAM are multiple and include prolonged reduction of pro-inflammatory cytokines, autophagy inhibition, and anti-angiogenesis. Here, we present an overview of the current preclinical (*in vitro *and *in vivo*) and clinical evidence supporting the role of CAM in cancer. Overall these findings justify further research with CAM in many tumour types, with multiple myeloma, lymphoma, chronic myeloid leukaemia (CML), and lung cancer having the highest level of evidence. Finally, a series of proposals are being made to further investigate the use of CAM in clinical trials which offer the greatest prospect of clinical benefit to patients.

## Introduction

Clarithromycin (CAM) (or 6-O-methyl erythromycin) is a member of the macrolide antibiotic family together with erythromycin, azithromycin, and roxithromycin. Unlike erythromycin, CAM is acid-stable, has a half-life (five hours) compatible with a twice-a-day administration, and a high and stable bioavailability. CAM has been available as a generic drug worldwide since 2005 [1].

The antimicrobial spectrum of CAM and other macrolides is slightly broader than that of penicillin and as such macrolides are a common substitute for patients allergic to penicillin. In addition, macrolides have been shown to be effective against *Legionella pneumophila*, *Mycoplasma pneumoniae*, *mycobacteria*, some *rickettsia*, and *Chlamydia trachomatis*, for which penicillin is not effective. CAM is also used for the eradication of gastric infection with *Helicobacter pylori*.

The antibacterial effect of macrolides is related to their capacity to inhibit protein synthesis in bacteria. They do so by binding to subunit 50S of the bacterial ribosome. Apart from their anti-bacterial activity, macrolide antibiotics have been long known to exhibit a wide range of other pharmacological effects such as the inhibition of mucus hypersecretion and the reduction of the levels of pro-inflammatory cytokines in patients with chronic inflammatory diseases [1].

## Dosage

CAM is usually administered, in tablet or oral suspension form, at a dose of 250 mg twice daily. A dose of 500 mg twice daily is recommended for severe infections or for *Helicobacter pylori *eradication. 500 mg three times daily is the highest dose approved and was used for *Helicobacter pylori* eradication before the advent of triple therapy [2]. In children the recommended dose is 7.5 mg/kg twice daily.

Anti-bacterial treatment with CAM is usually recommended for a duration of one to two weeks. Long-term (from 3 to 12 months) administration of CAM at the dose of 500 mg daily has been shown to be safe and effective in patients with chronic pulmonary disease or chronic sinusitis [3, 4]. Treatment for four years with 200 mg/day has been trialed in ten patients with diffuse panbronchiolitis with no adverse events except for transient mild diarrhoea which spontaneously resolved after a few days in one patient [5].

## Toxicity

CAM has a well-known toxicity profile thanks to its widespread use as an antibiotic. The most frequent and common adverse reactions are abdominal pain, diarrhoea, nausea, vomiting, and dysgeusia. Other common adverse reactions described are insomnia and rash. These adverse reactions are usually mild in intensity and resolve after treatment discontinuation. All other adverse reactions are uncommon or rare. In particular there are instances where effects on the central nervous system resulting in somnolence and confusion have been described in post-marketing surveillance.

Special caution should be employed when administering CAM to patients with impaired hepatic function or with severe renal insufficiency.

Because of the risk of prolonging the heart’s QT interval, CAM should not be given to patients with history of QT prolongation, cardiac arrhythmias, patients with hypokalaemia, or to patients treated with drugs that may result in QT prolongation and cardiac arrhythmias.

Recently, concerns have been raised about possible long-term effects of short CAM usage (e.g. daily for two weeks) on the risk of cardiovascular events and mortality in both patients with stable coronary heart disease and patients without heart disease [6, 7]. Longer intake was also associated with more cardiovascular events. The biological explanation supporting these associations is unclear even though it has been proposed that because clarithromycin may activate macrophages, it could lead to an inflammatory cascade resulting in more vulnerable plaques that over time may lead to acute coronary syndromes or sudden cardiac death by plaque rupture [8].

CAM inhibits cytochrome P450 3A4 (CYP3A4) which is involved in the hepatic metabolism of many drugs. It belongs to the intermediate CYP3A4 inhibition group of antibiotic macrolides, together with roxithromycin [9]. Co-administration of CAM with statins known to be metabolised by CYP3A4 (lovastatin and simvastatin) or with ergotamine or dihydroergotamine is contraindicated.

## Bioavailability

CAM is acid-stable and has demonstrated excellent and homogeneous bioavailability after oral administration. Following intestinal absorption, it undergoes a rapid first-pass hepatic metabolism. Its main metabolites are 14-(R)-hydroxyCAM, 14-(S)-hydroxyCAM, and N-desmethylCAM. 14-(R)-hydroxyCAM is an active metabolite also responsible for its anti-bacterial effect. CAM’s half-life is about five hours whereas 14-(R)-hydroxyCAM’s half-life is about seven hours [10–12].

## Pre-clinical evidence in cancer—*in vitro* and *in vivo *

Treatment with CAM alone was able to significantly delay the growth of Lewis lung carcinoma and reduce the number of tumour nodules in C57BL/6 mice, with a dose of 10 mg/kg/day being most effective in inducing antitumor effects [13]. In a B16BL6 melanoma model, CAM was administered at 50 mg/kg/day and this reduced the size of melanoma tumours by increasing apoptosis of tumour cells and significantly suppressing pulmonary metastases [14]. In a BALB/c murine B cell lymphoma cell line, CAM induced apoptosis which was demonstrated by the appearance of apoptotic bodies, DNA fragmentation, degeneration and detachment of the cells. No upregulation of Bcl-2 or FasL was seen; however, expression of tumour necrosis factor receptor 1 (TNFR1), caspase-3, -8, -9 suggested that apoptosis was induced through the tumour necrosis factor (TNF) system [15].

In addition to a direct effect on tumour cells, there is evidence that CAM can inhibit tumour-induced angiogenesis in mice [14]. Tumours from mice treated with 50–100 mg/kg CAM had significantly lower vessel density than tumours from the control group in the Lewis lung cancer model and the B16BL6 melanoma model [14, 16]. Endothelial tube formation was inhibited by CAM in a dose-dependent manner at concentrations greater than 10 microM *in vitro *[16].

Combination of approved anticancer agents with CAM also showed efficacy. In Lewis lung cancer bearing C57BL/6 mice, CAM administered seven days or more after vindesine or cisplatin significantly enhanced the effect of chemotherapy by increasing natural killer cell activity and CD8+ T cell cytotoxicity, by inducing the well-balanced expansion of helper T cell subsets recovering from immunosuppression caused by the chemotherapy. Intriguingly, this effect was not seen when CAM was administered immediately after chemotherapy [13]. In a melanoma tumour model, CAM potentiated the inhibition caused by cyclophosphamide, cisplatin, doxorubicin, or vindesine, possibly via an anti-angiogenic effect [17]. In rats bearing mammary adenocarcinoma, the death rate was decreased by administration of CAM combined with either carboplatin or cyclophosphamide, while either treatment alone did not affect the death rate. Interestingly, adding CAM to surgery as well as before surgery was beneficial [18]. *In vitro*, these rats’ mammary adenocarcinoma cells expressed lower matrix metalloproteinases-9 (MMP-9), transforming growth factor-beta (TGF-β), and tumour necrosis factor alpha (TNF-α) levels after CAM treatment [18, 19]. Interleukin-6 (IL-6) was transiently enhanced and decreased to basal levels thereafter, while the tissue inhibitor of metalloproteinase-2 (TIMP-2) expression was not affected [19]. CAM may modulate the tumour microenvironment and influence a tumour’s metastatic potential [20, 21]. Furthermore, in tumour-bearing rats, spleen cells from CAM treated animals expressed lower levels of TGF-β and IL-6 compared to untreated animals and had a stronger tumour neutralising activity [18].

There is evidence that CAM is a potent and continuous inhibitor of autophagy in both myeloma and chronic myeloid leukaemia cells. At clinically relevant concentrations (6 to 50 μg/mL) CAM inhibits lysosomal function (after fusion of the autophagosomes with the lysosomes) [22]. Thus, CAM could be a potential adjuvant to treatment modalities where autophagy is used by the tumour as an escape mechanism. The proteasome inhibitor bortezomib induces autophagy. The combination of CAM and bortezomib resulted in increased cytotoxicity compared to bortezomib alone [23]. Similar results were obtained using breast cancer cell lines. The combined treatment of bortezomib with CAM enhanced bortezomib induced cytotoxicity, despite the absence of apoptosis induction when CAM was used alone [24]. The apoptosis-inducing effect was further enhanced when vorinostat, a histone deacetylase 6 (HDAC6) inhibitor, was added to bortezomib and CAM [25]. Treatment of chronic myeloid leukemia cells with dasatinib, a tyrosine kinase inhibitor, also induced autophagy. As expected, combining CAM with dasatinib resulted in a significantly decreased percentage of living cells compared to dasatinib alone because of inhibition of late-stage autophagy. Even in a cell line with a clinically known dasatinib-resistant mutation, combination with CAM increased the sensitivity to dasatinib. Whereas clinically relevant concentrations of dasatinib alone had no effect on cell death, the combination with CAM achieved 32% cell death in these mutant cells [26]. These results are perfectly in line with clinical outcome in advanced CML patients treated with a combination of CAM and dasatinib (described below) [27].

## Human data

Numerous clinical trials have been reported with CAM in combination with other drugs in patients with multiple myeloma (MM) and Waldenström’s macroglobulinemia (WM) (Table 1). CAM has also been effective against some *H pylori*-associated cancers such as gastric mucosa-associated lymphoid tissue (MALT) lymphomas, either in a *H pylori *eradication regimen or as a single agent (Table 2). Finally, a handful of trials have demonstrated encouraging results in solid tumours unrelated to *H pylori *(Table 3).

### Multiple myeloma and Waldenström’s macroglobulinemia (Table 1)

The first report of the use of CAM in MM patients dates back to 1997. Durie *et al *reported at that time a greater than 50% response rate when 500 mg CAM was administered twice per day [28]. In this study, patients also received dexamethasone. Other groups using CAM as monotherapy could not confirm its potential in MM [29–32]. In a single-arm phase II study, the response to CAM and pamidronate was measured by changes in serum M protein and urinary M protein excretion. From the 15 evaluable patients, 12 (80%) did not show any response [30]. Furthermore, none of the 20 evaluable MM patients had a complete or partial response using standard response criteria after CAM monotherapy and none of the patients with rapidly progressing myeloma at the time of initiation of CAM displayed stable disease on treatment [31].

Similarly, in another study no response was observed in 35 MM patients receiving CAM monotherapy after a median time of eight weeks. In fact, MM progressed in 80% of cases, requiring other therapeutic approaches [32]. Also in the study by Musto, 33/38 evaluable patients (87%) did not show a response as measured by changes in M-component. However, in this study most patients received additional therapy with potential anti-myeloma activity of pamidronate (23 patients) and dexamethasone (ten patients)). Of the few patients showing a response, the strongest responders had received steroids during CAM therapy [29].

In contrast to the low efficacy as a monotherapy, the combination of CAM with steroids, with or without concomitant thalidomide or its analogues, resulted in high response rates, which is in line with the initial results of Durie [28] and the findings of Musto [29]. Vescio *et al *reported a 73% response rate during the 2001 annual meeting of the American Society of Haematology, when MM patients were treated with CAM (500 mg twice daily) and steroid therapy with or without thalidomide [33]. Coleman *et al *reported that the combination of CAM with dexamethasone and low-dose thalidomide in patients with MM or WM resulted in a 93% response rate [34]. Responses in half of the patients with WM who did not respond on thalidomide alone were also observed [35]. In another trial where omeprazole and aspirin were also administered, 83% of WM patients had a significant response [36].

A trial set up to determine the effect of adding CAM to low-dose thalidomide and dexamethasone on the response rate suggested that CAM potentiates dexamethasone’s effect. Addition of CAM to dexamethasone and thalidomide treatment led to a further tumour mass reduction at 16 and 20 weeks in patients whereas less than 50% response was seen upon induction therapy consisting of dexamethasone alone or in combination with thalidomide [37]. According to Niesvizky *et al *the addition of CAM would allow a lower dose of dexamethasone while maintaining a high response rate. Indeed, Morris *et al *reported an overall response rate of 96%, of which 89% had at least 50% reduction in paraprotein, in relapsed and refractory myeloma patients when they received CAM combined with low dose dexamethasone and low dose thalidomide [38].

Addition of CAM to treatment with dexamethasone and lenalidomide, one of the immune modulatory drugs (IMiDs) which are structural and functional analogues of thalidomide [39], also resulted in an objective response of 90% in treatment-naive MM patients in a phase II trial [40]. This treatment was very well tolerated and provided lasting responses. After long-term follow up (6.6 years) the median progression-free survival (PFS) was 49 months and the overall response rate increased to 93%, without an increased incidence of second primary malignancies [41]. This regimen is now known as the BiRd (Biaxin [CAM]/Revlimid [lenalidomide]/[low dose] dexamethasone) regimen. The addition of CAM allowed for a significant reduction of the dexamethasone dose and a higher complete remission response rate compared to a concomitant phase II study with only dexamethasone and lenalidomide treatment [40, 42].

It is possible that low-dose dexamethasone and not the CAM was responsible for the higher remission rates as the trials were not controlled [40, 43]. The value of adding CAM to this treatment regimen was therefore investigated by Gay *et al *in a case-matched study [44]. Patients in the BiRd regimen compared to patients in the Rd regimen had an increased complete or very-good-partial-response rate (73.6% versus 33.3%; P < 0.001), and a longer PFS (median values: 48.3 months versus 27.5 months; P = 0.044). Furthermore, a trend towards a better overall survival (OS) was noted with fewer infections and lower levels of dermatological toxicity. On the downside, more instances of thrombocytopenia and steroid-related myopathy were observed in the BiRd regimen compared to Rd. Both the frequency of neutropenia and the rate of thromboembolism were similar in both groups. Despite two toxic deaths in the BiRd group and none in the Rd group, a similar treatment discontinuation occurred in both groups because of adverse events [44].

Additional subgroup analysis indicated that only stage I/II patients benefited from the BiRd regimen, suggesting that CAM makes a difference only in early stage of disease. A randomised phase III study of BiRd versus Rd is planned to verify these results (personal communication with Dr Niesvizky and Dr Mark). Even in one patient with resistance to the Rd regimen, a response was achieved by adding CAM to Rd [45]. This ability of CAM has been recently confirmed by Ghosh who found this in ten (42%) out of 24 patients with MM based on their response when clarithromycin was added to Rd at the time of progression on Rd [46]. Since BiRd is so well tolerated, and transplant status did not show an effect on PFS or OS, BiRd may provide a less toxic alternative to transplantation [41]. Extending the combinatorial approach by addition of thalidomide to the BiRD regimen (T-BiRD) did not further improve the clinical outcome and was associated with increased non-haematologic toxicity [47].

Besides lenalidomide in the BiRD regimen, other IMiDs have also been used. Rossi *et al *reported the efficacy of CAM, pomalidomide, and dexamethasone (ClaPD) combination therapy for relapsed or refractory myeloma [47–49].

### Other haematological malignancies (Table 2)

Carella *et al *reported in 2012 that CAM could also improve clinical outcome of CML patients in combination with a tyrosine kinase inhibitor (TKI), either dasatinib or nilotinib. They serendipitously observed in one patient whose disease was not controlled with dasatinib that after treatment with CAM for otitis/pharyngitis the patient had a complete cytogenetic response. Three other CML patients who had become resistant to TKIs alone, also responded when CAM was added. The Bcr-abl/abl transcript levels fell in all four patients. In three of them, CAM was stopped for various reasons (not because of adverse events), resulting in a relapse and an increase in Bcr-abl/abl transcript levels. In two patients, Bcr-abl/abl transcript levels fell again when CAM was restarted. In the third patient, only the white blood cell count decreased upon recommencing CAM together with dasatinib and the transcript level continued to increase [27]. As of November 2013, all four patients are still alive, with some degree of cytogenetic and molecular remission. Two of them continue to receive CAM on alternate days (personal communication with Dr Carella).

Haematological malignancies associated with a bacterial infection might also benefit from CAM. Development and growth of gastric MALT lymphomas are often associated with a *H pylori *infection. Eradication of *H pylori *results in durable tumour regression in the majority of patients with low-grade gastric MALT lymphoma [50, 51]. In patients with high-grade gastric MALT lymphoma or primary gastric diffuse large B-cell lymphoma (DLBCL) durable complete remissions have been described in response to a combination of antibiotics which includes CAM [52–58]. Kuo *et al *assessed the efficacy of *H pylori *eradication therapy in patients with stage IE/IIE1 DLBCL of the stomach. The pCR rate was 58% and was not different between both *H pylori *positive *de novo *DLBCL and high-grade transformed MALT lymphoma [59]. Findings of this retrospective study were confirmed in a prospective trial in which 50% of patients with either *de novo *DLBCL or high-grade transformed MALT lymphoma achieved a complete response to *H pylori *eradication only [60].

In addition to non-Hodgkin’s lymphoma (NHL), there is also a case report of a patient with Hodgkin’s disease of the nodular sclerotic type who benefited from antibiotic treatment. CAM combined with another antibiotic, ciprofloxacin, resulted in an almost complete disappearance of the pulmonary lesions. The antibiotics were continued for another five months and more than two years later a complete remission was documented [61].

It is possible that the tumour responses described previously are only because of the antibacterial effect of CAM. Some reports suggest otherwise and propose a direct anti-tumour or immunomodulatory effect of CAM. Regression of low-grade MALT lymphoma of the rectum occurred after *H pylori *eradication treatment in a patient negative for *H pylori *[62]. A Japanese patient with both low-grade primary thyroid MALT lymphoma and gastric cancer with *H pylori *infection achieved a complete disappearance of the lymphoma after *H pylori *eradication. Immunostaining and PCR revealed no presence of *H pylori *in the thyroid MALT lymphoma [63]. Furthermore, a woman with MALT lymphoma of the duodenum and no evidence of *H pylori *infection was treated solely with long-term CAM and achieved a complete remission after six months of treatment [64]. In addition, a 38% overall response rate was achieved in 13 relapsed/refractory patients with extranodal marginal zone B-cell lymphoma (EMZL) after being exclusively treated with 500 mg of CAM twice a day. The long period between previous antibiotic therapies and trial enrolment together with the lack of evidence of bacterial infections made the authors suggest that the regression of EMZL was because of direct anti-tumour or immunomodulatory effect of CAM [65]. Moreover, two pulmonary MALT lymphoma patients benefited from long-term CAM treatment (200 mg/day). The first patient showed only a small reduction on conventional treatment (chemotherapy + radiotherapy) and received CAM to treat a sinobronchial syndrome. Regression was observed after a few months and the pulmonary MALT lymphoma was markedly reduced two years after initiation of CAM. The second patient did not receive conventional therapy, and *H pylori *eradication therapy did not succeed in eradicating *H pylori*. Despite that, the pulmonary MALT lymphoma almost disappeared after six months of CAM therapy [66]. More recently, in an abstract presented at ASCO in 2014, Saad *et al *confirmed that the effect of CAM was independent of *H pylori *eradication [67]. They randomised 60 patients with indolent non-Hodgkin lymphoma (NHL) to receive either CVP (cyclophosphamide, vincristine, and prednisone) or CVP plus CAM (500 mg twice a day D5-D18) every 21 days. MALT lymphoma patients were eligible after confirmation of *H pylori *negativity. Complete response rate, objective response rate, and two-year PFS were significantly better in the CAM arm with 32% versus 19%, 93% versus 70%, and 75% versus 46% respectively. Two-year OS was not significantly different between arms (90% versus 85%). A greater decrease in vascular endothelial growth factors (VEGF) after two cycles was observed in the CAM arm.

Ohe M *et al *described several cases in which it is considered likely that CAM contributed to a direct anti-tumour effect. In a case of follicular B-cell lymphoma, the lymphadenopathy regressed and the levels of soluble IL-2 receptor decreased following CAM treatment [68]. Combining CAM (800 mg/day) with a steroid, prednisolone, to treat a patient with diffuse large B-cell lymphoma resulted in a complete response after six months [69]. Also, 400 mg CAM, twice daily, combined with prednisolone and cyclophosphamide was successful in improving para-aortic lymphadenopathy, hepatomegaly, and ureteral dilatation at four months in a patient with follicular B-cell lymphoma. Soluble IL-2 receptor decreased. Further improvement was observed at eight months [70].

### Solid tumours (Table 3)

In addition to MM and lymphomas, there is evidence of anticancer activity of CAM in solid tumours. In a randomised trial in 49 patients with advanced lung cancer—42 with non-small cell lung cancer (NSCLC) and 7 with small cell lung cancer (SCLC)—reported in 1997, Mikasa *et al *found that the 25 patients treated with maintenance CAM (200 mg b.i.d.), commencing on the first post-discharge hospital visit and continuing for as long as they could tolerate it, had a significantly longer survival (median of 395 days) than patients who did not receive CAM (median of 256 days). The benefit seemed to only occur in NSCLC patients where survival time was almost doubled (median survival of 535 versus 277 days for NSCLC CAM patients versus NSCLC control patients). Patients receiving CAM were also able to stay at home for longer periods than those in the non-CAM group. They remained in good condition for a long period and had a good appetite; consequently, they gained weight during treatment [71]. These results were confirmed in a follow-up study in NSCLC from the same group and results were ascribed to reduced serum IL-6 levels after CAM treatment. Patients whose serum IL-6 levels decreased survived longer, irrespective of the histological type of disease, and the decrease in IL-6 statistically correlated with the increase in body weight [72]. Unfortunately, it seems that no further research has been published in NSCLC with CAM even though we have identified ten papers published in Japanese in the late 1990s and early 2000s.

CAM has also been shown to decrease acute inflammation after surgery. Both the incidence and duration of the postoperative systemic inflammatory response syndrome (SIRS) was significantly reduced in lung cancer patients receiving CAM compared to control [73]. Also the inflammatory response after mastectomy was modulated by CAM, as reflected by differences in temperature, heart rate, respiratory rate, and monocyte count between the treatment and control group. Furthermore, CAM had the additional benefit of decreasing the intensity and duration of pain from mastectomy [74].

## Clinical trials

On 3 November 2014, a search for ongoing trials using CAM as an anticancer agent in the treatment of cancer patients in clinicaltrials.gov revealed three trials in multiple myeloma (NCT01745588, NCT01559935, and NCT02248428) and three trials in *H pylori *associated malignancies (NCT01516606, NCT00327132, NCT01264822). On the same date, no trial was registered to treat cancer patients with other members of the macrolide antibiotic class currently available on the market (erythromycin, azithromycin, roxithromycin).

## Mechanism of action

There is a wealth of information describing the possible effects of CAM. When compiling information from the clinical trials and from the pre-clinical experiments, it is reasonable to hypothesise that several mechanisms of action explain the anticancer activity of CAM and that the mechanisms involved depend on the type of cancer and on the drugs it is combined with. The principal mechanisms of action are described below.

### Direct anti-tumour effect

Whether or not CAM has direct antineoplastic effects may depend on the tumour type. In a rat model, no direct effect on mammary adenocarcinoma was observed [18]. However, lymphoma tumour regression after CAM treatment independent of the eradication of *H pylori *suggests direct anti-tumour activity of CAM on lymphoma cells [64–66]. Indeed, CAM directly induces apoptosis in a murine B cell lymphoma cell line, via the TNF system [15]. Macrolides can also induce apoptosis of human peripheral blood derived lymphocytes [75]. In addition, in human blood mononuclear cells it is shown that CAM inhibits NF-κB activation induced by TNF-α [76]. NF-κB regulates gene expression, including upregulation of the anti-apoptotic genes bcl-2 and Bcl-xL. Furthermore, aberrant nuclear expression of NF-κB was generally detected in both the large cells and their low-grade counterparts within the *H pylori*-independent tumours [77]. This down-regulation of antiapoptotic genes by CAM (through NF-κB) might in part explain the effect of long-term use of CAM on tumour cells despite eradication of the infection or even in the absence of infection. The majority of DLBCL and gastric MALT cases show bcl-2 expression [78, 79]. In addition, some investigators have shown in mice that Bcl-xL might play a role in the pathogenesis of B-cell MALT lymphoma developed from chronic infection with *Helicobacter *species [80, 81]. At least in activated lymphocytes, the downregulation of Bcl-xL has been reported as the mechanism by which CAM induces apoptosis [82].

In addition to the NF-κB pathway, other pathways could also be involved. Some bacteria including, *H pylori*, can dysregulate the Wnt/β-catenin pathway. Given the contribution of the Wnt/β-catenin pathway in the carcinogenic process and the ability of some antibiotics (rapamycin, streptonigrin) to inhibit this, it is conceivable that CAM also interacts with this intracellular signalling pathway. To the best of our knowledge, no research has been done thus far on the influence of CAM on the Wnt/β-catenin signalling pathway. In this connection, Semino-Mora *et al *reported that β-catenin levels were decreased in the cytoplasm, the cell nuclei, and the mucin-associated cells while β-catenin levels within membranes increased in pseudomyxoma peritonei patients after *H pylori *eradication treatment containing CAM, providing potential protection against cell detachment, cellular invasion, and metastasis [83].

For the control of cellular proliferation, extracellular signal-regulated kinase (ERK) plays an important role [84, 85]. Modulation of ERK phosphorylation by CAM at physiologic concentrations retards cell proliferation of a normal bronchial epithelial cell line, by preventing cells passing from G1 into S phase of the cell cycle [86]. Several oncogenes can constitutively activate ERK, and uncontrolled activation of ERK can lead to malignant transformation [87, 88]. In NSCLC, ERK1/2 is activated and associated with advanced tumours [89]. The antitumor effect seen in NSCLC patients could therefore be partly mediated via modulation of ERK by CAM.

For multiple myeloma cells, IL-6 is an important cytokine with regard to proliferation, survival, drug resistance, and migration [90]. Since CAM inhibits IL-6 secretion [72], this might exert a direct anti-cancer effect in MM. However, inhibition of one single growth factor is unlikely to be sufficiently potent to obtain a clinical benefit for multiple myeloma [30–32].

### Synergism with approved anticancer drugs

#### Dexamethasone

Despite the poor clinical outcome after CAM as monotherapy in myeloma patients, CAM has an added value when combined with other treatment modalities such as thalidomide or its analogues, and steroids [44]. Such combinations have been used in more than ten different trials [33–35, 37, 38, 40, 41, 44, 47, 91–93]. As such, CAM augments the response to dexamethasone when given alone or in combination with thalidomide to patients with MM [37, 94].

However, the exact mechanism of this synergism is unknown. One possible explanation is that CAM slows the hepatic clearance of dexamethasone and other drugs used to treat MM as it does for other drugs metabolised by CYP3A4. Unfortunately, to the best of our knowledge, a direct comparison on the effect of CAM on dexamethasone metabolism has not been done and studies of interaction of CAM with other glucocorticoids (prednisolone and methylprednisolone) showed interaction with one but not the other [95]. There could be other reasons for this synergism. In one *in vitro *study, lymphocytes derived from CAM-treated asthma patients had a significant enhanced sensitivity to suppression by dexamethasone in the presence of CAM [96] suggesting a possible synergism in the modulation of immune cells subtypes. Furthermore, glucocorticoids (dexamethasone) induce apoptosis of lymphoid cells, which is attenuated by bcl-2. As CAM down-regulates bcl-2 expression by inhibiting NF-kB activation [76], CAM combined with glucocorticoids may synergise in inducing apoptosis.

In addition, both dexamethasone and CAM lead to suppression of inflammatory cytokines. Influencing inflammation to reduce tumour growth is discussed in more detail below. Glucocorticoids inhibit IL-8 through the glucocorticoid response element or the NF-κB binding site. CAM indirectly acts on the AP-1 binding site in the IL-8 promoter to repress IL-8 [97].

Recent findings indicate that glucocorticoids not only cause apoptotic cell death [98] but also induce autophagy resulting in tumour cell death in haematologic malignancies [99]. It has been suggested that adding other drugs inducing or targeting autophagy could lead to more tumour cell death, providing another explanation of the synergism between dexamethasone and CAM in MM [99].

To conclude on dexamethasone and CAM, the use of different mechanisms might underlie the additional value of combining dexamethasone and CAM.

#### Therapies inducing autophagy

The autophagy process provides tumour cells alternative sources of energy during periods of metabolic stress or starvation to maintain cellular homeostasis and survival [100]. Autophagy is also upregulated during extracellular matrix (ECM) detachment which is required for metastasis [101]. In MM cells, CAM contributed to the anti-tumour effect by halting the autophagy process at clinically relevant concentrations of 6–50 μg/mL [22, 23]. The combination of CAM and the proteasome inhibitor, bortezomib targets respectively the autophagy-lysosome system and the ubiquitin-proteasome system for protein degradation. This enhances endoplasmatic reticulum stress-mediated apoptosis by increased expression of the proapoptotic transcription factor CHOP (CADD153) in MM cell lines [23]. The additional benefit of CAM with TKIs could also be ascribed to the inhibition of autophagy used by cells to resist and escape treatment [26]. Many current cancer treatment modalities such as radiotherapy and chemotherapy also induce autophagy which promotes tumour dormancy and facilitates re-growth [100]. It can be hypothesised that CAM could overcome, or delay, resistance to conventional treatment via the inhibition of the autophagic response of cancer cells [26].

Concerns have recently been raised with regard to exploiting the autophagy inhibition mechanism that CAM seems to possess, especially in strategies that aim to also exploit the immune system. Scheduling of CAM treatment in relation to chemotherapy may be critical. It is possible that a very short course of CAM just at the time of chemo or radiation therapy might increase cell kill by inhibition of autophagy. However, allowing autophagy to take place may be critical for antigen presentation by dendritic cells [102], which occurs a few days after chemotherapy-induced cell kill. Thus CAM administration may be best avoided for a week or two following cell kill. On the other hand, autophagy blockade aids NK cell lysis, so that late institution of CAM treatment would serve this purpose [103]. The paper by Hamada *et al *emphasises the fact that CAM treatment was beneficial only when started one week post chemotherapy [13] and CAM treatment was instituted by Mikasa *et al* about a month after chemo and radiation therapy was over [71].

### Modulation of tumour environment

#### Immune modulating and anti-inflammatory action 

Macrolide antibiotics are known to have anti-inflammatory effects [1]. Anti-inflammatory effects relevant to cancer have been suggested by the capacity of CAM to reduce acute inflammation after mastectomy or lung cancer surgery [73, 74].

Cancer and chronic inflammation are closely intertwined. Chronic inflammation is known to drive tumour growth. Cytokines produced by immune cells, the tumour, and its environment are key regulators of the inflammatory response, able to either drive (pro-inflammatory) or suppress (anti-inflammatory) inflammation. CAM modulates ERK1/2 phosphorylation in bronchial epithelial cells and inhibits the activation of NF-κB and AP-1, two important transcription factors in inflammation [76, 97, 104, 105]. In this way CAM temporarily suppresses the production of the pro-inflammatory cytokines IL-1α, IL-1β, interleukin-1 receptor antagonist (IL-1RA), granulocyte-colony stimulating factor (G-CSF), granulocyte macrophage colony stimulating factor (GM-CSF), IL-6, IL-8 and TNF-α in human monocytes, eosinophils, monocytic leukemia cell lines, and bronchial epithelial cells [104–108]. A decrease in IL-6 was confirmed in serum of lung cancer patients [72]. In contrast, CAM enhanced IL-10 secretion by monocytes [106]. Inhibition of IL-8 by CAM has been shown to be temporary with levels increasing thereafter, to decrease again later to basal pre-inflammatory levels [105]. This immunomodulatory action of CAM and other macrolides resulting in an initial and short-lived increase in inflammation followed by a sustained decrease of cytokine production and secretion to normal levels is conceptualised as ‘resetting the circuits’ [1]. Furthermore, in chronic obstructive pulmonary disease (COPD) patients and patients with cystic fibrosis, a decrease of pro-inflammatory cytokines was seen in sputum and serum after CAM treatment [109, 110].

IL-8 is a chemokine produced by several cell types and attracts inflammatory cells to the site of infection. IL-8 is also a promoter of angiogenesis. It is believed to be partly responsible for the maintenance of chronic inflammation in gastritis caused by *H pylori *[111–113]. In order for IL-8 to be secreted in response to *H pylori *by a gastric cancer cell line (MKN45), the AP-1 and NF-κB binding sequences in the IL-8 promoter are required [114]. Also IL-8 secretion upon TNF-α and IFN-γ stimulation requires the same sequences [115]. CAM inhibits NF-κB activation and indirectly inhibits the AP-1 binding in the promoter region of IL-8, as found in epithelial, monocytic, and lymphocytic cell lines, and peripheral blood mononuclear cell (PBMC) [76, 97, 104]. As the mechanism for IL-8 transcription is similar in these cell types and the MKN45 gastric cancer cell line, and macrolides can enter eukaryotic cells without receptors the previous results can possibly be extrapolated to gastric cancer cells. This means that inflammation (TNF-α and IFN-γ) can maintain IL-8 secretion by gastric cancer cells and that this can become inhibited by CAM by inhibiting NF-κB activation and AP-1 binding in the IL-8 gene promoter region in gastric cancer cells. These findings suggest that the additional value of CAM in gastric cancers is dampening the chronic inflammatory tumour microenvironment.

CAM also modulates the immune system by other mechanisms. When combined with glucocorticoids and theophylline, it almost completely abolishes the survival effect of IL-5 on eosinophils [116]. In a mouse model of bleomycin-induced lung injury, CAM reduced the expression of the vascular adhesion molecule 1 (VCAM-1), affecting leukocyte migration to the site of inflammation [117]. *In vitro *after mitogen stimulation of T cells, CAM markedly inhibited T cell proliferation and IL-2 production [118]. The Th1 cytokines IL-2 and TNF-α were inhibited more markedly than the Th2 cytokines [119]. These *in vitro *results are in contrast to the results obtained in cystic lung fibrosis patients, in whom peripheral blood lymphocytes showed a sustained increased proliferation after CAM treatment. Prolonged CAM treatment in these patients showed reduction of cytokines in sputum and serum, concurrent with a switch from Th2 to Th1 type response measured by the IFN-γ/IL-4 ratio [110]. Williams *et al *reported that CAM decreased dose-dependently IL-4 secretion from human mononuclear cells stimulated with phorbol myristate acetate and ionomycin, such that the Th1/Th2 ratio increased [120]. If this effect of CAM holds true in the presence of a tumour, it could have an immune stimulatory effect against the tumour. In murine bone-marrow derived dendritic cells (DCs), CAM upregulated the expression of CD80, a T cell co-stimulatory molecule and decreased IL-6 production by DCs, without increasing IL-10 production. Stimulated naïve splenic T cells by these DCs showed reduced IL-2 levels [121]. Finally, IL-6 is known to stimulate the production of myeloid derived suppressor cells (MDSCs), a powerful class of immunosuppressive cells. CAM may therefore decrease MDSCs in the tumour environment though this is speculative at the moment.

#### Anti-angiogenesis

Besides influencing the secretory factors of epithelial cells and immune cells in the tumour microenvironment, animal models have shown that CAM also targets the tumour microenvironment by inhibiting tumour-induced angiogenesis [14, 16, 122]. The recent randomised trial by Saad *et al *in NHL indicates that adding CAM to a CVP regimen was able to reduce soluble vascular endothelial growth factor (sVEGF) level more than CVP alone. As these findings correlated with a clinical benefit in terms of response and PFS, they indicate that the effect of CAM involves the VEGF pathway [67].

CAM could have an impact on the same core cancer-associated biological processes as thalidomide and its analogues. These also modulate inflammatory cytokines, T cells, adhesion, expression of adhesion molecules, and angiogenesis [39]. To conclude this part on the mechanism of action, CAM acts on tumour-associated inflammation, angiogenesis, signal transduction pathways, growth signals, autophagy, and metastasis.

## Our Take

### Next steps

Based on the evidence summarised in Table 4 it is our contention that human clinical trials of CAM in certain types of cancer is warranted. The known pharmacokinetics, relatively low toxicity, low cost, and strong pre-clinical and clinical evidence make this an ideal candidate for repurposing.

#### Multiple myeloma

It seems that interest in CAM as an anticancer agent has taken off for MM and WM. However, no randomised trials have been performed and the most urgent next step in MM is to put CAM to the test in a randomised trial. This should happen soon thanks to the continuous efforts of Dr Tomer Mark, Dr Morton Coleman, Dr Ruben Niesvizky, and their colleagues at Weill Cornell Medical College and the New York Presbyterian Hospital (NY, USA). Also important would be to conduct pharmacokinetics/pharmacodynamics studies and other ancillary studies (cytokines, etc.) to document and understand how CAM works in the BiRd regimen.

#### *H pylori*-associated lymphomas

The value of CAM in eradicating *H pylori *was established 20 years ago. The complete and long-lasting responses in *H pylori*-associated malignancies after bacterial eradication are more recently observed. The most important step is to identify precisely patients who could benefit from a simple *H pylori *eradication in gastric DLBCL and MALT lymphoma. In 2001, Morgner reported that even high-grade MALT lymphomas, which are thought to have become independent of *H pylori*, could respond to *H pylori *eradication [56]. More than ten years later, clinicians started to identify patients who actually benefit from this simple approach and are providing reassuring data about the effectiveness of rescue strategies in case of failure of *H pylori *eradication [59, 60]. It will be important to gather additional data within clinical trials about the effectiveness and safety of this strategy.

#### Other B-cell lymphomas

As several authors have reported responses in lymphomas independent of *H pylori *[61–67], the most important action for further evaluating the potential of CAM in these lymphoma subtypes is to identify settings where CAM would be of help. Two situations come immediately to mind: when no other standard or experimental options are available or in patients with indolent disease in whom the balance between benefit and risk of cytotoxic treatment is unfavourable. In the latter situation, CAM could be tested as an alternative in a clinical trial.

#### Solid tumours

Finally, more research is desperately needed in solid tumours. To the best of our knowledge, there has been no attempt in Japan or anywhere else to confirm the survival benefit reported by Mikasa *et al *in 1997 in patients with NSCLC [71]. Several Japanese groups were active in the late 1990’s and early 2000’s but it seems that interest has waned since CAM became a generic drug in 2005. Currently, there are no clinical trials registered. We are aware of one trial under preparation by Prof. Albrecht Reichle (Regensburg, Germany) in patients with NSCLC where CAM will be administered together with a metronomic chemotherapy and pioglitazone (personal communication with Prof. Albrecht Reichle).

The evidence suggests that cancer types to take to human trials include:

- NSCLC in combination with standard first-line chemotherapy, based on Mikasa’s findings [71] and the documented benefit of using CAM in COPD patients [4]. However, because of the concerns of a possible negative impact of inhibiting autophagy on the role of the immune system [102, 103], it may be best to not administer CAM with chemotherapy (or radiotherapy). It might be best to give it one to two weeks following cell kill to allow optimal antigen presentation.
- SCLC in combination with chemotherapy, also based on COPD data and because the numbers of SCLC patients in the trial reported by Mikasa were too low to draw any conclusion, taking the caution mentioned above about the timing of CAM administration.
- CML in patients developing resistance to second generation TKIs as observed by Carella [27];
- Melanoma in the following settings:
  - In patients with BRAF-mutated tumours together with BRAF inhibitors to delay resistance as recent work have shown that resistance to BRAF inhibitors could be overcome by targeting autophagy in BRAF (V600E) melanoma [123], as well as in other BRAF (V600E)-mutated tumours [124]. CAM could be an alternative to chloroquine in targeting autophagy.
  - In patients progressing or not responding to checkpoint blockade agent based upon the immunomodulatory activity of CAM.
- Other advanced solid tumours resistant to conventional treatment. The use of metronomic schedule of chemotherapy together with CAM in these populations could be tested with the intention of synergising the immunomodulatory and anti-angiogenic effects of both interventions. The use of pharmacokinetics, biomarkers (especially immune and angiogenic ones), and dynamic imaging in such trials would be very informative to document the biological effects induced by this combination.

## Conclusion

Besides interfering with the protein synthesis, by which CAM exerts its antibacterial function, CAM also affects signalling pathways and transcription factors, drug pharmacokinetics, autophagy, immune function and inflammation, and angiogenesis. These features of CAM can be exploited to make tumour cells more prone to apoptosis and reduce escape mechanisms. As such, CAM can be used as a supportive drug in conventional cancer treatments and can even be used as a monotherapy for some tumours.

The anti-tumour activity of CAM has been demonstrated in tumour cell lines and mouse models. Clinical trials have confirmed the potential of CAM in NSCLC and lymphoma, even as a single treatment. In combination with other treatments CAM has proven effective in early stage multiple myeloma, and Waldenström macroglobulinemia. Larger randomised trials to establish CAM’s anti-tumour potential are needed as well as more insights into the pharmacokinetics of CAM in combination regimens. Furthermore, better identification of patients and tumours benefiting from CAM would be a leap forward in the use of this low-cost drug. Based on the available data, it is also worth attempting to extend the benefit of CAM to other tumour types such as CML, SCLC, melanoma, and breast cancer, and clinical trials to investigate these are warranted.

## Conflicts of interests

The authors declare that they have no conflict of interest. All the authors are associated with not-for-profit organisations that aim to repurpose drugs for oncology treatments.

## Author contributions

Primary authors: An MT Van Nuffel and Gauthier Bouche. Contributing authors: Vidula Sukhatme, Pan Pantziarka, Lydie Meheus, and Vikas P. Sukhatme. All authors have read and approved the final manuscript.

## Figures and Tables

**Table 1.** Clinical trials in multiple myeloma and Waldenström’s macroglobulinemia patients with CAM as cancer treatment.

Year	Cancer type	CAM combined with	Number of patients	Outcome	Reference
1997	MM	Antimyeloma agents including steroids, pamidronate	30 (23 evaluable)	26% CR 30% PR 26% SD 17% mixed	[28]
1999	MM	No other drugs (bisphosphonate allowed)	23 (20 evaluable)	0% OR 50% SD 50% PD	[31]
1999	MM	No other drugs	35	0% OR 80% PD	[32]
2001	MM	Steroids +/- thalidomide	11	73% OR 9% SD 18% PD	[33]
2001	MM	Pamidronate	20 (15 evaluable)	M protein production: - Decreased: one patient - Unsustained reduction: six patients - Stable: six patients - Rose: six patients	[30]
2002	MM or WM	Dexamethasone and thalidomide (low dose)	50 evaluable	13% CR 40% near CR 13% major response 27% PR	[34]
2002	MM	Pamidronate in 23 patients Dexamethasone in 10 patients	51 (38 evaluable)	13% reduction of M-component OR: one patient Minor response: two patients 87% no response or PD	[29]
2003	WM	Dexamethasone and thalidomide (low-dose)	12	25% PR 17% Monoclonal protein reduction >25%	[35]
2003	WM	Dexamethasone, thalidomide (low-dose), omeprazole and enteric-coated aspirin	12	25% near CR 25% major response 33% PR 17% minor response	[36]
2003	MM, stage II and III	Dexamethasone and thalidomide (low-dose)	9	Median drop in the paraprotein level was - Before CAM 26% at week 8 - After CAM (started at week 8) 52% at week 16 55% at week 20	[37]
2008	MM	Dexamethasone, lenalidomide, aspirin, omeprazole and trimethoprim/sulfamethoxazole	72 (69 evaluable)	39% CR (of which 31% stringent CR) 14% near CR 21% very good PR 17% PR 6% minor response Outcomes were compared to matched controls in 2010 with the following results: CR or very good PR: 74% versus 33% Median TTP: 48.3 versus 27.5 months Median PFS 48.3 versus 27.5 months Outcomes were updated in a 2013 paper: 43% CR (of which 31% sCR) 25% very good PR 25% PR	[40, 41, 44, 93]
2008	MM (relapsed and refractory)	Dexamethasone (low dose), thalidomide (low-dose or dose escalation) and biphosponates (pamidronate or zoledronate)	30 (28 evaluable)	18% CR 39% good PR 32% PR 7% Minimal response 4% SD	[38]
2014	MM	Dexamethasone, lenalidomide and thalidomide (T-BiRd regimen)	26 (25 evaluable)	8% stringent CR 36% VGPR 36% PR 20% SD	[47]

Abbreviations: MM = multiple myeloma; WM = Waldenström’s macroglobulinemia; CR = complete response; pCR = complete pathologic remission; PR = partial response; OR = objective response; SD = Stable disease; PD = progressive disease; TTP = Time-to-progression; PFS = progression-free survival; = CAM as monotherapy; > = higher; ASIP = atypical serum immunofixation patterns, VGPR = very good partial response, sCR = strigent complete response.

**Table 2.** Clinical trials and published cases reporting use of CAM as cancer treatment in haematologic malignancies other than multiple myeloma and Waldenström’s macroglobulinemia.

Year	Cancer type	CAM combined with	Number of patients	Outcome	Reference
1997	Rectal MALT lymphoma	Omeprazole, amoxicillin	1	Regression confirmed pathologically	[125]
1999	Low grade rectal MALT lymphoma	Lansoprazole, amoxicillin	1 (*H pylori*negative)	Complete regression	[62]
1999	Gastric MALT lymphoma	Either with amoxicillin or with tetracycline	34 (28 *H pylori*positive)	*H pylori*-positive patients: 50% CR 29% PR 36% no response *H pylori*-negative patients: 100% no response	[126]
2000	Low-grade gastric MALT lymphoma	Omeprazole, amoxicillin	7	100% complete histologic regression	[127]
2001	Low-grade gastric MALT lymphoma	Omeprazole, metronidazole	97	79% CR 9% PR 11% no response	[128]
2001	High grade B-cell gastric lymphoma	Omeprazole, metronidazole	8	87% CR 13% PR	[56]
2001	High grade gastric MALT lymphomas	Amoxicillin, omeprazole	16	62% CR	[53]
2001	Primary high grade gastric DLBCL	Amoxicillin, lansoprazole	1	no evidence of lymphoma on histology	[55]
2001	Low grade gastric MALT lymphoma (stage IE and IIE)	Amoxicillin, lansoprazole	44 (34 *H pylori*-positive patients)	43% complete histological regression (56% within *H pylori*-positive patients)	[51]
2001	Gastric DLBCL	Amoxicillin, omeprazole	1	Regression	[58]
2003	Gastric DLBCL	Lansoprazole, metronidazole	1	CR still ongoing after 5.5 years	[57]
2003	Hodgkin’s Disease of the nodular sclerotic type, stage IIb	Ciprofloxacin	1	Almost complete disappearance of the pulmonary lesions after three months. Treatment continued for another five months. More than two years later: complete remission	[61]
2003	Low-grade Thyroid MALT lymphoma	Lansoprazole, amoxicillin	1	Complete disappearance of lymphoma after gastric cancer with *H pylori*infection treated by *H pylori*eradication therapy	[63]
2004	Gastric marginal zone B cell lymphoma of MALT (stage I)	Omeprazole and metronidazole or amoxicillin	95 (90 evaluable)	62% CRM 18% minimal residual disease 12% PR 4% no change 2% PD	[50]
2005	Early-stage, gastric low-grade transformed MALT lymphoma Early-stage, gastric high-grade transformed MALT lymphoma	Amoxicillin, omeprazole Amoxicillin, omeprazole	34 (30 patients with eradicated *H pylori*) 24 (22 patients with eradicated *H pylori*)	80% CR in patients where *H pylori*was eradicated 64% CR in patients where *H pylori*was eradicated	[54]
2006	Duodenal MALT lymphoma	No other drugs	1	CR	[64]
2008	B-cell gastric lymphoma (high grade)	Omeprazole and amoxicillin	1 61	Complete response 69% complete response	[52]
2010	Extranodal marginal zone B-cell lymphoma (EMZL) (relapsed/ refractory) (stage IE and IV)	No other drugs	13	15% CR 23% PR 31% SD 31% PD	[65]
2010	Pulmonary MALT lymphoma	No other drugs	2	regression	[66]
2011	Follicular B-cell lymphoma	No other drugs	1	Regression of lymphadenopathy Decrease in soluble IL-2 receptor	[68]
2012	Advanced CML	Tyrosine kinase inhibitor	4	- 25% Complete haematologic response -100% of patients had Bcr-abl/abl transcript level reduction	[27]
2012	*H pylori* positive gastric DLBCLs (*de novo* and MALT) stage IE/IIE1	Amoxicillin, omeprazole	*De novo* DLBCL: -chemo: 30 - antibiotics: 16 DLBCL (MALT): 34	*De novo* DLBCL - 69% pCR DLBCL (MALT): - 56% pCR	[59]
2012	*H pylori* positive gastric DLBCL (*de novo*and MALT)	Tinidazole, metronidazole, omeprazole	16 DLBCL of which: -*De novo*: 11 - MALT: 5	50% CR 19% PR 31% PD	[60]
2012	DLBCL (stage IV)	Prednisolone	1	CR after six months	[69]
2013	Follicular B-cell lymphoma	Prednisolone, cyclophosphamide	1	Improvement of the para-aortic lymphadenopathy, hepatomegaly and ureteral dilatation and decrease in soluble IL-2 receptor after four months which improved further after eight months	[70]
2014	NHL	Cyclophosphamide, vincristine and prednisone (CVP)	60 (55 evaluable) randomised in CVP and CVP+CAM	CVP versus CVP+CAM CR: 19% versus 32% (p = 0.04) ORR: 70% versus 93% (p = 0.04) 2-year PFS: 46% versus 75% (p = 0.02) 2-year OS: 85% versus 90% (p = 0.64)	[67]

Abbreviations: MALT = mucosa-associated lymphoid tissue; CML = chronic myeloid leukaemia; DLBCL = diffuse large B-cell lymphoma; NHL = non-Hodgkin lymphoma; CR = complete response; pCR = complete pathologic remission; PR = partial response; OR = objective response; SD = Stable disease; PD = progressive disease; OS = overall survival; PFS = progression-free survival, IL-2 = interleukin-2.

**Table 3.** Clinical trials in patients with solid tumours with CAM.

Year	Cancer type	CAM combined with	Number of patients	Outcome	Reference
1997	Lung stage IIIa, IIIb, IV (NSCLC & SCLC)	No other drugs (Prior chemo or radiation therapy or both)	49 - CAM: 22 NSCLC & 3 SCLC - no CAM: 20 NSCLC & 4 SCLC	NSCLC: increased median survival time SCLC: no significant difference in median survival time	[71]
2000	Breast cancer (mastectomy)	No other drugs	54 -28 CAM -26 no CAM	Reduced change in temperature, heart & respiratory rate and monocyte count after surgery	[74]
2001	Lung stage IIIa, IIIb, IV (NSCLC)	No other drugs (Prior chemo or radiation therapy or both)	47 - CAM: 33 patients - No CAM: 14 patients	Not indicated	[72]
2004	Lung stage I, II, IIIa	-control: Flomoxef -CAM: flomoxef + CAM	- Control: 16 patients - CAM: 10 patients	Significant reduced duration of SIRS	[73]

Abbreviations: NSCLC: non-small-cell lung cancer; SCLC = small-cell lung cancer; CAM = clarithromycin; SIRS = Systemic inflammatory response syndrome.

**Table 4.** Summary of the pre-clinical and clinical evidence available for the use of CAM as an anticancer agent, per cancer type.

Cancer type	In Vitro	In Vivo	Case report/Trial
Multiple Myeloma	[22, 23]		[28–34, 37, 38, 40, 41, 44, 47, 93]
CML	[26]		[27]
Waldenström’s macroglobulinemia			[34–36]
Lung (NSCLC)	[16]	[13]	[71–73]
Breast	[19, 24, 25]	[18, 21]	
Melanoma		[14, 17]	
Lymphoma	[15]		[27, 50–70, 125–129]

**Table 1.** Clinical trials in multiple myeloma and Waldenström’s macroglobulinemia patients with CAM as cancer treatment.

Disease	Targets	Drug combination (with selected references)
**Breast cancer**	Pro-inflammatory cytokines, monocytes … During and after surgery	NSAIDs [18], aspirin [19], beta-blockers [20]
**NSCLC**	Pro-inflammatory cytokines, monocytes … During and after surgery and/or radiotherapy	NSAIDs [13, 14], aspirin [21], cimetidine [22], beta-blockers [14]
**NSCLC**	Angiogenesis and endothelial cells	Metronomic [6, 23] or maintenance [1, 2] chemotherapy, aspirin [19, 24]
**All solid tumours, highly inflammatory (e.g. high neutrophils to lymphocytes Ratio)**	Pro-inflammatory cytokines, monocytes, dendritic cells, T-cell trafficking …	NSAIDs [13], metronomic chemotherapy [23]
**Melanoma and other immunogenic tumours**	Immune cells of the tumour environment (effector T-cells, Myeloid-derived suppressor cells, regulator T-cells, macrophages)	Immune checkpoint inhibitors [25], cellular immunotherapies [26]

## Appendix 1. Repurposing Drugs in Oncology (ReDO)—clarithromycin as an anti-cancer agent–supplementary material

### Introduction

The following drugs warrant further investigation in combination with clarithromycin (CAM), both in pre-clinical studies and potentially in clinical trials. These combinations, listed in Table 1, have been selected on the basis of existing pre-clinical and clinical experience in each of the indications. Haematologic malignancies are not listed as the research agenda with clarithromycin is more advanced in these diseases. All of these proposed combinations are expected to display relatively low toxicity and yield low cost and are generally available agents.

### Higher priority agents

The agents listed below have a high degree of clinical evidence of efficacy and are currently either in clinical use in oncology or are currently being investigated in clinical trials. They have been selected as potential agents to be used in combination with CAM and existing standard of care treatments. Note that these drugs are not listed in order of priority.

- *Maintenance chemotherapy*: combining CAM with a maintenance regimen could be of interest especially in lung cancer. Pemetrexed maintenance is now standard of care in non-squamous, non-small cell lung cancer (NSCLC) [1, 2], and adding CAM to an already effective maintenance regimen could turn out to be beneficial as like in Mikasa’s trial where he used CAM as a maintenance regimen as well [3]. Lung cancer frequently develops in patients with COPD which is characterised by a risk of infectious exacerbation and systemic inflammation. The antibiotic and anti-inflammatory properties of macrolides are certainly attractive in this patient population [4] knowing that a trial of prophylactic antibiotics—with a macrolide (roxithromycin) and a fluoroquinolone—in SCLC patients have demonstrated a clear benefit of this strategy [5]. It would be worth testing this hypothesis for other cancers characterised by a strong chronic inflammatory response.
- *Metronomic chemotherapy*: in heavily pre-treated patients, a combination of CAM with metronomic chemotherapy could be particularly attractive in advanced or metastatic lung cancer after failure of one or several lines of chemotherapy. A trial in patients with advanced NSCLC has shown that metronomic vinorelbine as a single agent was able to induce responses and stabilisation in heavily pre-treated patients [6] and Mikasa’s trial actually used CAM in a typical metronomic schedule [3]. Both interventions have an effect on the tumour microenvironment which could be an advantage in heavily pre-treated patients with refractory tumours. Tolerability of both CAM (reviewed in this article) and metronomic chemotherapy [7] has been generally good and the combination of the two is not expected to cause significant problems as long as CAM and chemotherapy drug(s) are not competing with CYP3A4 for their respective metabolism.
- *Non-steroidal anti-inflammatory drugs (NSAIDs) such as diclofenac*: in most cancers, the systemic inflammation response becomes predominant at some point and is associated with a poor prognosis [8]. Efforts are being made to target specific components of this systemic response. In addition to celecoxib which has been used in many cancer experiments and trials [9], other anti-inflammatory drugs have the potential to dramatically modify the inflammatory response leading amongst other things to modification of the sub-types of immune cells populating the environment of tumours [10]. A marker study validating this concept in a few patients might be an important prelude to justifying such a trial with survival endpoints.
- *Drugs in the perioperative period*: in addition to the use of CAM and anti-inflammatory drugs in patients with advanced disease, data indicate that both CAM [11, 12] and anti-inflammatory drugs [13] are able to affect the inflammatory and pro-angiogenic response induced by cancer surgery. A perioperative intervention combining these two might prove even more effective as has been shown experimentally by combining NSAID and beta blockers [14].
- *Immune checkpoint inhibitors*: even though this would be an expensive strategy because of the cost associated with the use of anti- cytotoxic T-lymphocyte-associated protein 4 (CTLA4) and anti-programme cell death ligant-1 (PD-(L)1) antibodies, it would make sense, thanks to the prolonged effect of CAM on numerous pro-inflammatory cytokines [15]. Non-responders to immune checkpoint blockade or those showing minimal response may show improved response. The effect of adding CAM to anti-PD-(L)1 antibodies could be tested in mouse models in concomitant or sequential combination with anti-PD-(L)1 antibodies.
- *BRAF-inhibitors*: In patients with BRAF-mutated tumours, CAM could be proposed together with BRAF inhibitors to delay resistance as recent work have shown that resistance to BRAF inhibitors could be overcome by targeting autophagy in BRAF (V600E) melanoma [16], as well as in other BRAF (V600E) mutated tumours [17]. CAM seems an interesting alternative to chloroquine in targeting autophagy.
